# Adaptive amino acid substitutions enable transmission of an H9N2 avian influenza virus in guinea pigs

**DOI:** 10.1038/s41598-019-56122-6

**Published:** 2019-12-24

**Authors:** Liu Lina, Chen Saijuan, Wang Chengyu, Lu Yuefeng, Dong Shishan, Chen Ligong, Guo Kangkang, Guo Zhendong, Li Jiakai, Zhang Jianhui, Luo Qingping, Zhang Wenting, Shang Yu, Wang Honglin, Zhang Tengfei, Wen Guoyuan, Zhu Jiping, Zhang Chunmao, Jin Meilin, Gao Yuwei, Shao Huabin, Zhao Zongzheng

**Affiliations:** 1 Institute of Animal Husbandry and Veterinary Sciences, Hubei Academy of Agricultural Sciences, Wuhan, China; 2 Institute of Military Veterinary, Academy of Military Medical Sciences, 666 West Liuying Road, Changchun, 130122 Jilin China; 3 Institute of Mountainous Area Research, College of Veterinary Medicine, Hebei Agricultural University, Baoding, 071001 Hebei, China; 4 College of Veterinary Medicine, Huazhong Agricultural University, Wuhan, China; 5 Hubei Engineering Research Center of Viral Vector, Wuhan university of Bioengineering, 430415 Wuhan, China

**Keywords:** Influenza virus, Viral evolution, Viral pathogenesis

## Abstract

H9N2 is the most prevalent low pathogenic avian influenza virus (LPAIV) in domestic poultry in the world. Two distinct H9N2 poultry lineages, G1-like (A/quail/Hong Kong/G1/97) and Y280-like (A/Duck/Hong Kong/Y280/1997) viruses, are usually associated with binding affinity for both α 2,3 and α 2,6 sialic acid receptors (avian and human receptors), raising concern whether these viruses possess pandemic potential. To explore the impact of mouse adaptation on the transmissibility of a Y280-like virus A/Chicken/Hubei/214/2017(H9N2) (abbreviated as WT), we performed serial lung-to-lung passages of the WT virus in mice. The mouse-adapted variant (MA) exhibited enhanced pathogenicity and advantaged transmissibility after passaging in mice. Sequence analysis of the complete genomes of the MA virus revealed a total of 16 amino acid substitutions. These mutations distributed across 7 segments including PB2, PB1, PA, NP, HA, NA and NS1 genes. Furthermore, we generated a panel of recombinant or mutant H9N2 viruses using reverse genetics technology and confirmed that the PB2 gene governing the increased pathogenicity and transmissibility. The combinations of 340 K and 588 V in PB2 were important in determining the altered features. Our findings elucidate the specific mutations in PB2 contribute to the phenotype differences and emphasize the importance of monitoring the identified amino acid substitutions due to their potential threat to human health.

## Introduction

H9N2 avian influenza virus (AIV) was first detected from turkeys in 1966 in the United States of America and transmitted to a variety of mammalian species including swine, dogs, weasels, mink, bats and humans^1–6^. The first case of a human infection with H9N2 was reported in 1998, in China^7^. Seroprevalence studies have shown that the antibodies to H9N2 were higher in humans who had direct exposure to poultry, particularly in occupational poultry workers, suggesting that infections with H9N2 commonly occur^5,8,9^. H9N2 AIV can also replicate in mammals without prior adaption, and its ongoing mammalian adaption poses a significant public health risk^10–12^. Notably, H9N2 AIV is presumed to be the donor of the internal genes of several prevalent reassortant AIVs, such as H5N1, H7N9 and H5N6 viruses^13–17^. Airborne transmission contributes to the dissemination of H9N2 worldwide and has enabled H9N2 to transmit efficiently among poultry, but no human-to-human transmission of H9N2 has been observed. The airborne transmissibility of H9N2 in mammals remains unknown^18^.

Several experimental methods have been used to explore the transmissibility of AIVs in mammals. In general, the methods rely upon mammalian adaptation through serial passage, reassortment between an AIV and a human influenza virus, point mutations in genes, or a combination of the above^19^. A highly pathogenic strain of H7N1 avian influenza virus became capable of airborne transmission in mammals after 10 serial passages^20^. An avian H5N1 virus acquired airborne transmission in guinea pigs after receiving genes from 2009/H1N1virus^21^. The HA and PB2 genes are important for cross-species transmissions of AIVs^22^. An H5N1 virus modified by site-directed mutagenesis and subsequent serial passage in ferrets became airborne transmissible^23^.

The capacity of AIVs to transmit among mammals appears to require multiple viral features, such as human receptor binding, increased polymerase activity and high thermostability of HA^20,21,23–25^. Human receptor binding specificity, specifically leucine (L) at position 226 in HA receptor binding site, is critical for direct transmission of avian H9N2 viruses in ferrets^10^. Increased viral polymerase activity mediates adaptation of AIVs to a mammalian host^26^. The high thermostability of HA facilitates H5 AIV transmission via respiratory droplets in mammals^27^.

The transmission of AIVs to mammals appears to acquire human receptor binding preference^28–30^. Y439 (A/Duck/Hong Kong/Y439/1997), G9 (A/Chicken/Hong Kong/G9/1997), G1 (A/quail/Hong Kong/G1/97) and Y280 (A/Duck/Hong Kong/Y280/1997) are four different H9N2 poultry lineages. The G1 and Y280 poultry lineages are usually associated with both avian and human receptor binding affinity and could potentially transmit between mammals^8^. Mice have been widely applied to study mammalian adaptation of AIVs. Serial passage of AIVs in mammals can result in adaptive changes that confer enhanced pathogenicity and transmissibility in mammals^31–33^. Although the pathogenicity and transmissibility of H9N2 AIVs have been characterized previously^33–35^, the molecular features that account for H9N2 airborne transmissibility in mammals are not clear. In the current study, a Y280-like H9N2 virus transmitted among guinea pigs after mouse adaption. To explore which gene-specific mutations contribute to altered phenotype, we generated recombinant and mutant viruses using reverse genetics technology.

## Materials and Methods

### Ethics statement

The ethics statement was described in our previous work^36^. Briefly, all animal studies were conducted in strict accordance with the Guidelines of Animal Welfare of World Organization for Animal Health and the protocols approved by the Hubei Provincial Animal Care and Use Committee (approval number SYXK 2016-0004).

### Viruses

The wild type H9N2 virus used in this study was isolated from chickens in 2017, in China, and named A/chicken/Hubei/214/2017 (abbreviated as WT). A single amino acid substitution in PB2 was generated by using A Quick Change XL Site-Directed Mutagenesis Kit (Stratagene, La Jolla, CA). The WT and MA were the parental viruses. We used the WT as the backbone to generated the recombinant reassortant viruses (WT-PB2_MA_, WT-PB1_MA_, WT-PA_MA_, WT-NP_MA_, WT-HA_MA_, WT-NA_MA_, WT-NS_MA_) and the mutant viruses (WT-PB2_340K_ and WT-PB2_588V_) as described previously^37^. The recombinant reassortant viruses each contained one gene from the MA virus and the mutant viruses each was a single amino acid substitution in the PB2 of WT. The viruses were propagated in 9-day-old specific pathogen free (SPF) embryonated eggs and stored at −80 °C.

### H9N2 adaptation in mice

The mouse-adapted H9N2 virus was derived from series of sequential lung-to-lung passages of the WT virus in mice as described previously^32^. Briefly, groups of three five-week-old female BALB/c mice were anesthetized with ether and intranasally inoculated with 50 μL of a 10^6^ EID_50_ solution of the WT virus. Lungs were harvested and homogenized in 0.7 mL of PBS at 3 dpi. The supernatants were subsequently used to inoculate three naive mice. The infected mice died at 3 dpi at the fourth passage. The mouse-adapted virus (MA) was isolated from the homogenized lung tissue supernatants using 9-day-old SPF embryonated eggs for subsequent use in pathogenicity and transmissibility studies.

### Sequence analysis

The viral gene sequences were acquired as described in our previous work^36^. In brief, viral RNA was extracted from allantoic fluid using TRIzol reagent (Invitrogen) and reverse transcribed into cDNAs using the primer Uni12 (5′-AGC RAA AGC AGG-3′) primers, an RT reagent kit and viral genes were amplified using a PCR kit (Takara, Japan) according to the manufacturer’s protocol. The PCR products of the eight segments of the viruses were amplified by PCR using specific virus primers as described by Hoffmann *et al*.^38^. The PCR products were purified and sequenced by Sangon Biotech Company. Amino acid substitutions between the WT and MA viruses were identified. All the sequence data were analyzed with the SeqMan program (DNASTAR, Madison, WI). All reference sequences used in this study were obtained from the National Center for Biotechnology Information (NCBI) GenBank database.

### Receptor binding specificity assay

The receptor-binding specificities of the WT and MA viruses were determined by HA assays with 1% chicken red blood cells (cRBCs) as described in our previous work^36^. For the HA assay, sialic acid residues were enzymatically removed from cRBCs by incubating the cells with 50 mU of *Vibrio cholerae* neuraminidase (VCNA, Roche, San Francisco, CA) at 37 °C for 1 h, followed by resialylation using either α2-,6-N-sialyltransferase or α2-,3-N-sialyltransferase (Sigma-Aldrich, St. Louis, MO) at 37 °C for 4 h. The sample was then washed two times with phosphate-buffered saline (PBS), centrifuged at 1500 rpm for 5 min each time, adjusted to a final working concentration (1%) with PBS, and stored at 4 °C. For the HA assay, viruses were serially diluted 2-fold with 50 μL of PBS and mixed with 50 μL of a 1% RBC suspension in a 96-well plate. HA titers were determined after 1 h at 4 °C.

### Cell culture and growth curves

The virus growth curve experiment was performed as described in our previous work^39^. Madin-Darby canine kidney (MDCK) cells were obtained from the American Type Culture Collection (ATCC) and maintained in Dulbecco’s modified Eagle’s medium (DMEM; Invitrogen, Carlsbad, CA, USA) supplemented with 10% fetal bovine serum (FBS; Gibco, Auckland, New Zealand). The growth kinetics of the WT and MA viruses were determined by inoculating MDCK cells at a multiplicity of infection (MOI) of 0.001 50% tissue culture infectious dose (TCID_50_) per cell. One hour after inoculation (hpi), the cells were washed twice with PBS, and fresh medium supplemented with 1 μg/mL tosyl phenylalanyl chloromethyl ketone (TPCK) and trypsin (Sigma, St. Louis, MO, USA) was added. The supernatants were sampled at 12, 24, 36, and 48 hpi. The virus titers were determined by calculating the lg TCID_50_/mL in MDCK cells. The TCID_50_ values were calculated according to the method of Reed and Muench.

### Mouse experiments

Mouse experiments were performed as described in our previous work^40^. Groups of five six-week-old female BALB/c mice (Merial Vital Laboratory Animal Technology Company, Beijing, China) were anesthetized with ether and intranasally inoculated with 50 μL of 10^6^ EID_50_ solution of the test virus or PBS. The weight loss and mortality of mice in these groups were monitored daily for 14 days. Mice that lost >30% of their original body weight were humanely euthanized.

### Guinea pig experiments

Guinea pig experiments were performed as described in our previous work^36^. Hartley strain female guinea pigs weighing 300 to 350 g (Merial Vital Laboratory Animal Technology Company, Beijing, China), confirmed to be seronegative for influenza viruses prior to the experiment, were used in these studies. In the transmission studies, groups of three guinea pigs were anesthetized with ether and intranasally inoculated with 300 μL of 10^6.0^ EID_50_ solution of the test virus and housed in a cage placed in an isolator. The next day, three naive guinea pigs were individually paired and cohoused with an infected guinea pig for the direct contact transmission studies, and another naive guinea pig was housed in a wire frame cage adjacent to the infected guinea pig for the aerosol transmission studies. The distance between the cages of the infected and aerosol-contact guinea pigs was 5-cm. To monitor virus shedding, nasal washes were collected from all animals at 2, 4, 6, and 8 dpi and titrated.

### Statistics analysis

Statistically significant differences were determined using one-way analysis of variance (ANOVA) with GraphPad Prism software (San Diego, CA, USA). All assays were run in triplicate, and the data are representative of at least 3 separate experiments. The error bars indicate the standard deviation.

## Results

### The adapted H9N2 virus exhibits enhanced pathogenicity

**Figure 1:**
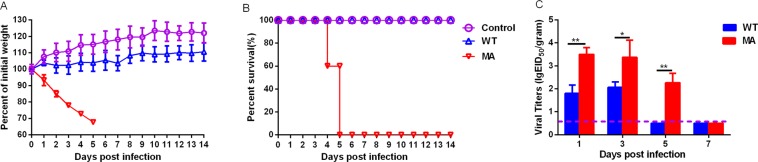
Pathogenicity in mice. Five mice per group were intranasally inoculated with 10^6.0^ EID_50_ of PBS, WT or MA. (**A**) Mouse body weights were monitored daily for 14 days. The values are the average scores of the overall body weight loss with respect to the initial body weights, ±standard deviations (SDs). (**B**) The survival percentages were calculated by observing the infected mice. (**C**) Lungs were collected from mice inoculated with 10^6.0^ EID_50_ WT or MA at 1, 3, 5 and 7 dpi (n = 3), virus titers were determined in 9-day-old SPF embryonated eggs (EID_50_/gram). Briefly, the lung tissues were weighed, and 0.1 grams of each tissue was placed into 1 ml of PBS containing 100 U/ml penicillin, to make 10% weight/volume lung homogenates (*P < 0.05; **P < 0.01).

We studied the pathogenicity of the MA virus in mice. Mice inoculated with the MA virus rapidly lost more than 30% of their original weight and succumbed to death at 5 dpi (Fig. 1), its MDL_50_ was 10^4.5^ EID_50_/mL. In contrast, the WT-inoculated mice experienced no substantial body weight loss and had nonlethal infections (Fig. 1A,B). These results show that a series of lung-to-lung passages of the H9N2 virus resulted in substantially increased virulence in mice.

Additionally, we also tested the viral titers of WT and MA in the lungs of the mice. In the MA-infected mice, the titers were 10^3.5^EID_50_/gram, 10^3.4^EID_50_/gram and 10^2.3^ EID_50_/gram at 1, 3 and 5 dpi respectively which were 10 fold higher than those of WT (*0.01 < P < 0.05; **P < 0.01; n = 3). No virus shedding was detected in the lungs of the WT-infected mice at 5 and 7 dpi (Fig. 1C). These results suggest that MA showed advantageous growth properties in the lungs of infected mice compared to WT.

In summary, based on the results of mice studies, the MA virus exhibited increased virulence and advantageous growth ability compared to the WT virus.

### The adapted H9N2 virus replicates to higher titers in MDCK cells

**Figure 2:**
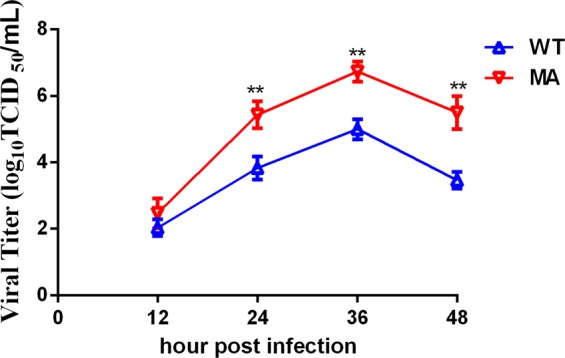
Characterization of viral growth kinetics in MDCK cells. Growth kinetics of the WT and MA viruses. MDCK cells were infected with the WT or MA virus at an MOI of 0.001 TCID_50_ per cell and treated with 1 mg/mL TPCK. At the indicated hpi, the virus titers in the supernatants were determined in MDCK cells. The reported values are the means and standard deviations of three independent experiments (**P < 0.01).

To evaluate the replicative capacity of the WT and MA viruses, we tested the growth curve of WT and MA in MDCK cells. The virus titers of WT and MA peaked at 10^6.7^ TCID_50_/mL and 10^5^ TCID_50_/mL at 36 hpi, respectively (Fig. 2). The virus titer of MA was 10 fold higher than that of WT (**P < 0.01, n = 3), suggesting MA replicate more efficiently than WT.

### The adapted H9N2 virus display human and avian receptor binding affinity

**Figure 3:**
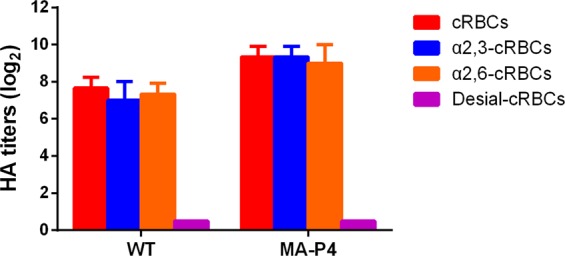
Agglutination activities of the WT and MA viruses in various red blood cells. Four types of chicken red blood cells (cRBCs) were used: a, cRBCs. b, α-2,3 cRBCs (treated with VCNA and resialylated with α-2,3 glycans). c, α-2,6 cRBCs (treated with VCNA and resialylated with α-2,6 glycans). d, desialylated(Desial) cRBCs (treated with VCNA). The HA titers showed the agglutination activities of the WT and MA in the four types of cRBCs. The reported values are presented as the means and standard deviations of three independent experiments.

Human receptor-binding specificity is an important factor for cross-species transmission of AIVs^41,42^. We thus measured the receptor binding specificity of the two viruses as previously described^36^. Briefly, the receptor binding affinity was determined by evaluating the ability of WT and MA to agglutinate four types of cRBCs. cRBCs contain avian and human receptors, while cRBCs treated with VCNA contains no receptors (desialylation-cRBCs), and resialylated cRBCs contained either human (α2,6-cRBCs) or avian (α2,3-cRBCs) receptors. The HA titers represent 3 separate experiments. The results showed that the WT and MA viruses bind to both avian and human receptors (Fig. 3).

### The adapted H9N2 virus transmits in guinea pigs

**Figure 4:**
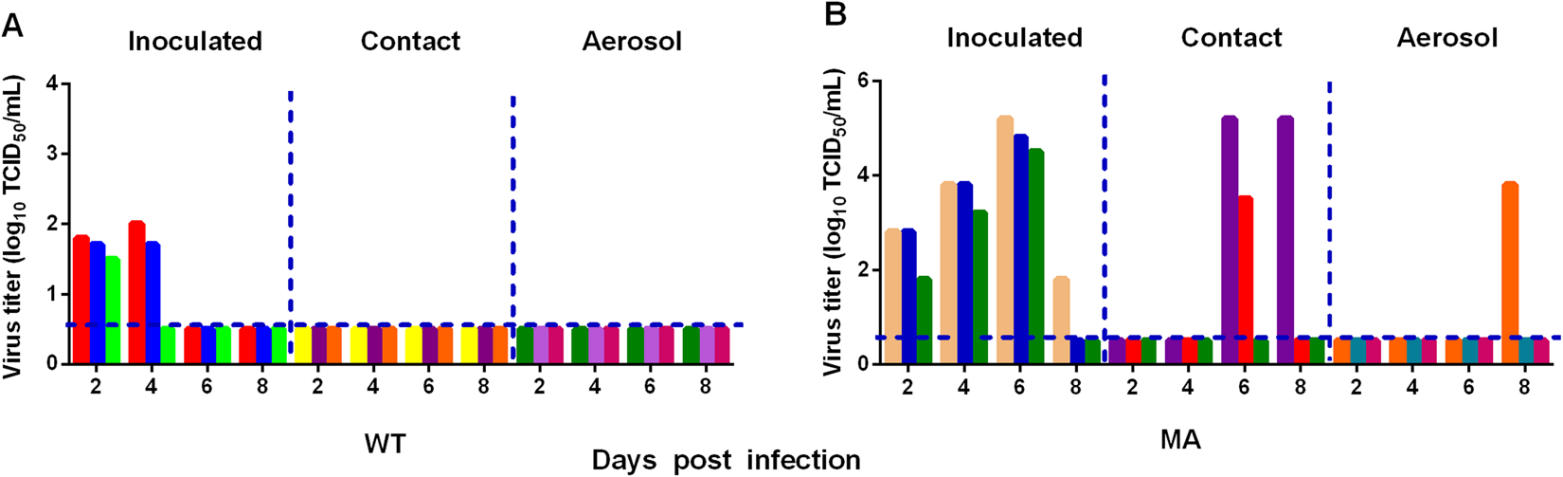
Horizontal transmissions of the viruses in guinea pigs. Groups of three guinea pigs seronegative for influenza viruses were inoculated with 10^6.0^ EID_50_ of the test viruses. The next day, the three inoculated guinea pigs were individually cohoused with a direct-contact guinea pig; in addition, an aerosol contact guinea pig was housed in a wire frame cage adjacent to that of the infected guinea pig. The distance between the cages of the infected and aerosol-contact guinea pigs was 5 cm. Nasal washes were collected from all animals for virus shedding detection every other day beginning on day 2 after the initial infection. Each color bar represents the virus titer in an individual animal. The dashed lines indicate the lower limit of virus detection.

To explore the impact of mouse adaptation on the transmissibility of the MA virus, we next measured the transmissibility of WT and MA in guinea pigs following the same procedures as we previously reported^36,43^. The WT viruses were only detected in the infected group, and no viruses were detected in the contact group or in the aerosol contact group, indicating that no virus transmission occurred (Fig. 4A). The MA viruses transmitted to 2 direct contact guinea pigs and 1 aerosol contact guinea pig (Fig. 4B). These findings demonstrate that the MA virus has acquired transmissibility in the guinea pig model after mouse adaptation.

### Sequence analysis in the adapted H9N2 virus

The molecular basis for the increased virulence and transmissibility was investigated by sequencing the complete genomes of WT and MA viruses. Sixteen amino acid substitutions were identified as shown in Table 1, and these mutations were distributed across 7 segments of the influenza genome. These included 3 changes in PB1 proteins, 4 changes in PA proteins, 2 changes in each the PB2, NP, HA and NS1 proteins and a single change in NA protein.

**Table 1:** Amino acid substitutions in the MA virus.

Segment	Position	WT	MA
PB2	340	R	K
	588	A	V
PB1	48	K	Q
	368	V	I
	628	M	L
PA	343	S	A
	356	K	R
	423	V	I
	554	V	I
NP	217	V	I
	239	V	M
HA	235	M	Q
	254	K	R
NA	72	R	K
NS1	127	N	T
	216	T	P

### Mutations in PB2 enable H9N2 to transmit among guinea pigs

**Figure 5:**
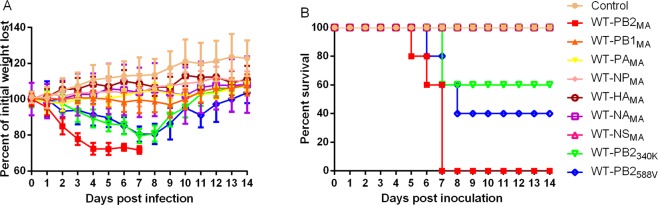
The pathogenicity of the rescued viruses. Five mice per group were intranasally inoculated with 10^6.0^ EID_50_ of the rescued viruses. (**A**) Mouse body weights were monitored daily for 14 days. The values are the average scores of the overall body weight loss with respect to the initial body weights, ±standard deviations (SDs). (**B**) The survival percentages were calculated by observing the infected mice.

**Figure 6:**
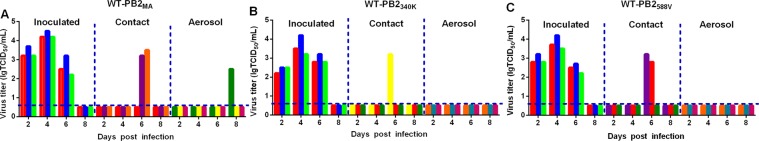
Assessment of amino acid substitutions in PB2 on transmission in guinea pigs. (**A**–**C**) Transmissibility of WT-PB2_MA_, WT-PB2_340K._ and WT-PB2_588V._ Nasal washes were collected from all animals for virus shedding detection every other day beginning on day 2 after the initial infection. Each color bar represents the virus titer in an individual animal. The dashed lines indicate the lower limit of virus detection.

To confirm which gene-specific mutations contributed to the increased pathogenicity and transmissibility of the MA virus, we used the WT virus as the backbone to generate a panel of recombinant viruses, each containing one gene from the MA virus. The pathogenicity of the recombinant viruses was studied in mice. The recombinant virus containing the PB2 gene from MA (WT-PB2_MA_) exhibited increased virulence, and the body weight loss and mortality of the infected mice were comparable to those of the MA-infected mice. Whereas the other recombinant viruses WT-PB1_MA_, WT-PA_MA_, WT-NP_MA_, WT-HA_MA_, WT-NA_MA_, WT-NS_MA_ caused no substantial body weight loss and nonlethal infections (Fig. 5). In addition, we also evaluated the transmissibility of the recombinant viruses. The WT-PB2_MA_ transmitted to 2 direct contact guinea pigs and 1 aerosol contact guinea pig (Fig. 6A), but the other recombinant viruses transmitted to neither the direct contact groups or the aerosol contact groups (data not shown).

Two amino acid substitutions, 340 K and 588 V, were identified in the PB2 gene of the MA virus. Therefore, we generated variant viruses contained a single amino acid substitution in PB2 in WT backbone (WT-PB2_340K_ and WT-PB2_588V_). The two variants also displayed increased pathogenicity than the recombinant viruses, with the exception of the WT-PB2_MA_ virus (Fig. 5). In guinea pig study, both WT-PB2_340K_ and WT-PB2_588V_ transmitted to direct contact guinea pigs, but no virus detected in the aerosol contact guinea pigs (Fig. 6B,C). These results suggest the combination of 340K and 588V in PB2 contributed to the aerosol transmissibility of the MA virus.

## Discussion

H9N2 AIVs pose a potential threat to public health. In this study, a Y280-like H9N2 virus displayed increased pathogenicity and transmissibility after serial passage in mice. We found that PB2-340K in combination with PB2-588V contributed to the altered features.

Influenza A virus can infect a variety of animal species. The receptor binding specificity of AIV is recognized as an important factor in interspecies transmission^22,24^. The HA gene of influenza A virus contains receptor binding sites and determines the receptor-binding specificity. AIVs isolates with 226-Leu(L) and 228-Gly(G) (H3 numbering) in HA have been reported to prefer both avian and human receptors^41,44,45^. The loss of glycosylation at residue 158 in the HA was also shown to be responsible for H5N1 AIV binding to human receptors^46^. In this study, there was no difference in receptor binding specificity between the WT and MA viruses with the *in vivo* results for the WT-HA_MA_ virus that didn’t show difference from the WT, suggesting the HA was not involved in the altered phenotype.

H5N1, H7N9, H9N2 and H5N6 AIVs have been reported to occasionally break the species barrier to infect humans, but they have not been able to disseminate among humans^23,46–48^. The major reason is their limited airborne transmissibility among humans. Previous studies found that ferrets and guinea pigs adaptation enabled AIVs to transmit in mammals^20,33^. In our previous study, mouse adaption could not enable the H5N6 to transmit in the guinea pig model^32^, but it enabled airborne transmission of the H9N2 in this study. We suppose the reason for its airborne transmissibility after mouse adaption might be correlate with its avian and human receptors binding affinity.

Several previous works have studied H9N2 adaptation to chickens or mammals. The HA-363K and PA-672L enabled H9N2 airborne transmission among chickens^49,50^. Passaging H9N2 in swine increased its replication and transmissibility^51^. The PB1-577E increased pathogenicity of H9N2 in mice^52^. The HA1-227P, HA2-46E and NP-434K enabled H9N2 contact transmission in guinea pigs^33^. The loss of glycosylation at 166 in HA and PB2-627K were also shown to increase virulence of H9N2 in mice^53^. Previous studies found that the PB2 gene of H9N2 played an important role in mammals^54^, they had identified PB2-404L, PB2-235N PB2-147L and PB2-627K enhanced pathogenicity of H9N2 in mice^34,55,56^. The PB2-E627K substitution was consistently found to mediate mammalian adaptation and a known determinant of pathogenicity and host specificity of AIVs^26,57,58^. However, the previous identified amino acid changes in H9N2 were not observed in this study. The PB2-R340K, PB2-A588V, PA-K356R, PA-S343A, NP-V239M and NS1-T216P identified in this study have been previously implicated in increasing virulence of other subtypes of AIVs^59–63^. PA-R356K was considered as a unique signature of H7N9 viruses with bird-to-human transmissibility and was also found to enhance viral polymerase activity, replication and pathogenicity in mammals^60,61^. The PA-S343A mutation was found to increase the polymerase activity and virulence of a low-pathogenic H5N1 influenza virus^62^. The NP-V239M and NS1-T216P mutations were defined as signature amino acids of H7N9 viruses isolated from confirmed human cases in Shenzhen of China^63^. PB2-R340K and PB2-A588V were previously found to increase viral polymerase activities, replication and pathogenicity of H10N8 and H7N9^59^. In the present study, we also found the substitutions PB2-R340K and PB2-A588V in combination enabled H9N2 airborne transmission among guinea pigs. These findings further highlight the need for persistent surveillance efforts to detect the emergence of H9N2 isolates with the identified amino acid substitutions in PB2.
